# Lifting the veil on disrespect and abuse in facility-based child birth care: findings from South West Nigeria

**DOI:** 10.1186/s12884-019-2188-8

**Published:** 2019-01-22

**Authors:** Macellina Y. Ijadunola, Ezeomu Audrina Olotu, Olaitan O. Oyedun, Stanley O. Eferakeya, Faith I. Ilesanmi, Ayotomiwa T. Fagbemi, Omowunmi C. Fasae

**Affiliations:** 1 Department of Community Health, College of Health Sciences, Obafemi Awolowo University, P.O. Box 2064, Ile-Ife, Nigeria; 2 Department of Community Health, Obafemi Awolowo University Teaching Hospitals Complex, Ile-Ife, Nigeria; 3 Obafemi Awolowo University, Ile-Ife, Nigeria

**Keywords:** Maternal health, Respectful maternity care, Disrespect and abuse, Childbirth, Africa

## Abstract

**Background:**

Eliminating disrespect and abuse in health care facilities during childbirth could be a contributory factor in improving pregnancy outcomes and avoiding preventable illnesses and deaths. This study aims to provide evidence of disrespect and abuse in this community in order to create awareness about its occurrence.

**Methods:**

A cross-sectional survey was carried out on 384 recently delivered women who visited the postnatal and immunization clinics of a primary and tertiary health facility in Ile-Ife. Information was sought about awareness of disrespect and abuse, prevalence and forms of disrespect and abuse, and opinions on improvements which can be made in maternity services. Univariate analysis was used to summarise the data.

**Results:**

About half of the respondents were in their fourth decade of life and had tertiary education. Overall, the majority (98.4%) of respondents agreed that it was their right to be treated with respect and dignity during childbirth while about one-fifth (19%) had ever experienced some form of disrespect and abuse. The commonly identified forms of disrespect and abuse were: non-dignified care (12.8%), discrimination (8.1%), a detention and abandonment (6%). However, the majority (81%) of the respondents did not have any suggestions for improvements in delivery services.

**Conclusions:**

Although most of the respondents knew it was their right to be treated with respect, some reported that they had experienced disrespect and abuse during childbirth in varying forms. The evidence from this survey draws attention to the need for interventions to address the health system factors hindering health service utilization.

## Background

In March 2010, the United States Agency for International Development (USAID) funded Translating Research into Action (TRAction) project, called for a meeting of public health and human rights governmental and non-governmental organisations who were active in maternal health issues to review the subject of respectful and disrespectful birth care including abusive maternal care [1]. This was motivated by the understanding that disrespect and abuse during childbirth not only involves human rights violations but is also one of the barriers to the utilization of a skilled birth attendant at delivery [1–3]. Over the last twenty years, efforts have gone into improving the number of deliveries taken by a skilled birth attendant in order to reduce morbidity and mortality. These efforts have yielded positive results as the proportion of deliveries attended by a skilled birth attendant in developing countries have increased from 56% in 1990 to 62% in 2012 however, 800 women and 7700 newborns still die daily from complications during pregnancy, delivery and postpartum period [4].

For a long time, the focus regarding barriers to skilled care has been on access to care until it was realised that improved access doesn’t necessarily translate to active use. Thus, shifting the focus to quality of care in terms of not just the skill of health workers, but also with regards patient’s rights and their perceptions of the quality of care they received [2, 3]. On-going research reveal that pregnancy outcomes are also linked to the experience of care and to reduce preventable illnesses and deaths, health care delivery should not only involve good infrastructure and skill but also proper delivery in a gentle, caring way and with the right attitude [4].

The World Health Organization (WHO) has defined a quality of care and prepared a framework for providing optimum care for mothers and newborn around the time of pregnancy, delivery and postpartum because they realized that adequate care in this period contributes maximally to saving lives [4]. According to WHO, Standards for improving the quality of maternal and newborn care in health facilities [4]:

“The quality of care for women and newborns is therefore the degree to which maternal and newborn health services (for individuals and populations) increase the likelihood of timely, appropriate care for the purpose of achieving desired outcomes that are both consistent with current professional knowledge and take into account the preferences and aspirations of individual women and their families”.

The framework divides quality of care into two parts: the provider’s provision of care and the patient’s experience of care. Embedded in the patient’s experience of care is a section on standards of care [4] with one of the domains stating that “women and newborn receive care with respect and preservation of their dignity” [p.3]. This is meant to address all forms of abuse, discrimination, neglect, detainment, and services denial [2, 4].

There have been reports of disrespect and abuse during labour and delivery from around the world and this occurrence has been defined “as interactions or facility conditions that local consensus deems to be humiliating or undignified, and those interactions or conditions that are experienced as or intended to be humiliating or undignified” [5]. The seven categories of disrespectful maternity care which have been highlighted by Browser and Hill in their landmark paper on the analysis of disrespect and abuse are: physical and verbal abuse, non-consented clinical care, non-dignified care, discrimination, non-confidential care, abandonment and detainment in a health facility. These may be due to behavioural and structural factors. Evidence from literature suggests that behavioural factors may be linked to learned behaviour from pre-service training, the belief that healthcare workers are acting in the best interest of patients, and a possible lack of commitment to ethics and respect for human right concerns of their patients [1, 6, 7]. Furthermore, many authors have also noted that structural challenges including under-staffing, poor pay and poor facility space, may lead to disrespectful maternity care [2]. Disrespect and abuse have been recorded in sub-Saharan Africa. Findings from Kenya revealed that 20% of the women polled reported some form of disrespect and abuse during their maternity care ranging across six categories which includes: non-consented care, abandonment and detainment, non-dignified care, physical abuse and non-confidential care. They perceived they were humiliated when receiving care during their labour and deliveries [8]. In Ghana, a study on exposure to disrespectful maternal care among final year student midwives had 72% opining that maltreatment during labour was a problem and they reported that the occurrence was more in government facilities compared with private facilities. About 80% of respondents also reported that the way women were treated during their labor and delivery influenced their choice of a delivery place, a probable reason why many women decided to deliver at home [9].

Disrespectful and abusive care has also been recorded in Nigeria. Okafor, Ugwu, and Obi in 2012 noted that 98% of their respondents reported one form of disrespectful and abusive care in their last delivery. This ranged from non-consented and non-dignified care to abusive care and abandonment and the commonest was the non-consented care and physical abuse. Non-consented care was carried out for procedures such as episiotomies, blood transfusions and caesarean sections and physical abuse included being beaten, slapped, restrained and tied as well as incidences of sexual abuse by health workers [10]. In Abuja, north-central Nigeria, Bohren et al. interviewed patients, health workers, and other hospital staff who observed patients being slapped, threatened, shouted at and physically restrained while on admission. Although some viewed this type of behavior as unacceptable, however, few of them believed actions like these were necessary in order to motivate the women to comply with care to ensure healthy outcomes. Some alleged that slapping was necessary to give the woman “more strength to push the baby out” [7].

In the face of evidence which proves the presence of disrespect and abuse in maternity care, the concept of Respectful Maternal Care (RMC) has evolved from the safe motherhood initiative and supported by USAID along with the White Ribbon Alliance. It is “an approach centred on the individual, based on principles of ethics and respect for human rights, and promotes practices that recognize women’s preferences and women’s and newborns needs” [11].

A few studies have shown that some of the victims of disrespectful and abusive maternity care sometimes accept the abuse they get as rightfully deserved, acceptable, and may not recognize any infringement on their fundamental human rights. Some regard this kind of behavior as “normal” and come to expect it [1, 7, 9].

In spite of available evidence of disrespect and abuse of women in facility-based childbirth care, there are few interventions geared towards reducing disrespectful and abusive care and promoting respectful maternal care in this environment. There is the need to create awareness by providing data and proof of its occurrence. This study is aimed at providing empirical evidence to support the growing trend of reports from other research sources carried out in other parts of the country and in the West African Sub-region. Studies done in Nigeria are few and none of them provide information about the South West geopolitical zone. It is also aimed at investigating the existence and magnitude of disrespect and abuse of women during childbirth from the client’s perspective in health facilities in Ile-Ife, south West Nigeria.

## Methods

This was a descriptive cross-sectional survey. Respondents were recruited for the survey at the postnatal and immunization clinics of a primary and a tertiary public health facility in Ile-Ife, Osun state and these were women who booked for antenatal care and had facility-based delivery in the selected facilities. Using the WINPEPI software’s formula for estimating a proportion and a prevalence of 20% from a similar study in Tanzania, at a 95% confidence level, 5% level of significance and a 20% attrition rate, a minimum sample size of 303 was determined. For a robust analysis, 400 questionnaires were eventually administered. As women presented in the immunization or postnatal clinic, they were recruited by trained research assistants until the sample size of 400 was met. Of the 400 questionnaires administered, 384 were returned completed while 16 were discarded due to mothers not completing the interview and hurrying to leave the facility after receiving clinical care giving a response rate of 96%. Inclusion criteria were all women who had recently had a baby in the last three months while women who did not deliver in the health facilities were excluded. Interviews were conducted for eligible respondents who were serially recruited over a period of two weeks in the order of their registration at the selected clinics.

A pre-tested interviewer-administered semi-structured questionnaire was used to collect information on awareness of respondents about disrespect and abuse among women who had facility-based childbirth and the prevalence of this disrespect and abuse. Prevalence was defined as “ever experience” of disrespect during childbirth for each respondent interviewed. Questions were also asked about the different forms of disrespect and abuse they had experienced as well as their opinion on areas for improvement in maternity services. The questionnaires were administered by trained interviewers and the questions were translated into the Yoruba language and back-translated into the English language to ensure accuracy in the portrayal of the intended meaning. Interviews were conducted in the Yoruba language for those who could not speak the English language. Participation was voluntary, confidentiality was assured and verbal consent was obtained from respondents before interviews were conducted.

Data were field edited then entered and analysed using SPSS version 17. Univariate analysis to generate frequency tables was performed for socio-demographic characteristics, awareness about disrespect and abuse in facility-based childbirth, the prevalence of disrespect and abuse, the different forms of disrespect and abuse they experienced and their opinion regarding areas of improvement in maternity services. Responses to open-ended questions were grouped into similar categories, transformed into quantitative variables then frequencies were determined.

## Results

A total of 384 women were recruited into the study. Their ages ranged from 15 years to more than 50 years with more than half of them (51%, *N* = 177) in the 30–39 year age group. Another half of them (52.7%, *N* = 202) had a tertiary education while almost half (49.2%, *N* = 189) were either artisans or traders. More than 7 in 10 of them were Christians and more than 8 in 10 were Yoruba (Table 1).

**Table 1:** Socio-demographic characteristics

Variable	Frequency (384)	Percentage (%)
Age-group(years)		
15–20	12	3.5
21–29	144	41.5
30–39	177	51.0
40–49	13	3.7
> 50	1	0.3
Education		
No formal	4	0.8
Primary	18	4.7
Secondary	123	32.1
Tertiary	202	52.7
Post-tertiary	37	9.7
Occupation		
Artisan/Trader	189	49.2
Professional	110	28.6
Corper/Student	37	9.6
Housewife/None	45	11.7
Clergy/Police	3	0.8
Religion		
Christianity	306	79.9
Islam	75	19.5
Others	3	0.8
Ethnicity		
Yoruba	335	87.2
Hausa	6	1.6
Igbo	30	7.8
Others	13	3.4

As shown in Table 2, more than 9 in 10 of them said that it was not culturally acceptable to be disrespected or abused during childbirth. About the same proportion agreed that it was their right to be treated with respect and dignity while 11% (*N* = 44) of them reported knowing someone who had been treated disrespectfully or abused during childbirth.

**Table 2:** Awareness of disrespect and abuse

Variable	Yes (%)	N0 (%)
Is it culturally acceptable to be disrespected and abused during childbirth?	13 (3.4)	371 (96.6)
Do you have a right to be treated with respect and dignity during childbirth?	378 (98.4)	6 (1.6)
Do you know anyone treated with disrespect and abuse during childbirth?	44 (11.1)	340 (88.5)

Table 3, which was developed from the question “Have you ever been disrespected or abused during maternity care?” revealed that one-fifth (19%, *N* = 73) of respondents had ever experienced disrespect and abuse during childbirth. In Table 4, respondents identified the different forms of disrespect and abuse they had experienced. The commonest form was the non-dignified care (12.8%, *N* = 49) followed by discrimination (8.1%, *N* = 31) and the least identified was physical abuse (1.6%, *N* = 6).

**Table 3:** Prevalence of disrespect and abuse

Variable	Frequency (384)	Percentage (%)
Yes	73	19.1
No	311	88.9

**Table 4:** Identified forms of disrespect and abuse

Variable	Frequency (384)	Percentage (%)
Physical abuse		
Yes	6	1.6
No	378	98.4
Non-consented care		
Yes	18	4.7
No	366	95.3
Non-confidential care		
Yes	20	5.2
No	364	94.8
Non-dignified care		
Yes	49	12.8
No	335	87.2
Abandonment		
Yes	23	6.0
No	361	94.0
Discrimination		
Yes	31	8.1
No	353	91.9

Different forms of physical abuse were experienced by 6 out of the 384 respondents which include being pinched or beaten (50%, N = 3) respectively while 33.3%, *N* = 2 were either slapped or sexually abused (Table 5).

**Table 5:** Forms of physical abuse experienced during the last pregnancy

Variable	Frequency (6)	Percentage (%)
Slapped		
Yes	2	33.3
No	4	66.7
Pinched		
Yes	3	50.0
No	3	50.0
Beaten		
Yes	3	50.0
No	3	50.0
Sexual abuse		
Yes	2	33.3
No	4	66.7

The forms of non-consented care are shown in Table 6, as experienced by 18 out of 384 respondents, and was more commonly about lack of information on the care received (77.8%, *N* = 14).

**Table 6:** Forms of non-consented care experienced during last labour and delivery

Variable	Frequency (18)	Percentage (%)
Procedures/Operations		
Yes	5	27.8
No	13	72.2
Lack of information about care		
Yes	14	77.8
No	4	22.2

Table 7 describes the non-confidential care received among respondents. The commonest was about asking private questions in the presence of other patient or healthcare workers not directly providing service to respondents (60%) while the least was taking delivery in the presence of other patients and their relatives (15%).

**Table 7:** Forms of non-confidential care experienced during last labour and delivery

Variable	Frequency (20)	Percentage (%)
Examined without a screen or partition		
Yes	7	35.0
No	13	65.0
Unduly exposed during an examination		
Yes	7	35.0
No	13	65.0
Asked private questions in the presence of others		
Yes	12	60.0
No	8	40.0
Delivery in view of the public		
Yes	3	15.0
No	17	85.0

Table 8 described the different forms of abandonment and detention and 7 in 10 of the respondents who experienced disrespect and abuse said they were left unattended during delivery and were denied necessary care which they expected in form of prompt response when they requested for medical attention or the constant presence of healthcare workers in the delivery room. Out of the 23 women who responded to question on being detained due to inability to pay hospital fees, 2 said yes, they had been detained.

**Table 8:** Forms of abandonment and detention experienced during last labour and delivery

Variable	Frequency (23)	Percentage
Left unattended to		
Yes	18	78.3
No	5	21.7
No encouragement during delivery		
Yes	6	26.1
No	17	73.9
Denied necessary care		
Yes	17	73.9
No	6	26.1
Detained for lack of payment		
Yes	2	8.7
No	21	91.3

In Table 9, the non-dignified care which occurred most was being shouted at (59.2%) while the use of harsh tone and words was a close second (49%). The least form was being threatened (6.1%).

**Table 9:** Forms of non-dignified care experienced during last labour and delivery

Variable	Frequency (49)	Percentage (%)
Shouted at		
Yes	29	59.2
No	20	40.8
Insulted		
Yes	22	44.9
No	27	55.1
Threatened		
Yes	3	6.1
No	46	93.9
Use of harsh words/tone of voice		
Yes	24	49.0
No	25	51.0
Intentionally humiliated		
Yes	7	14.3
No	42	85.7

Regarding forms of discrimination, Table 10 showed that perceived discrimination occurred most commonly due to economic status (38.7%) and least commonly due to HIV/AIDS status (6.5%).

**Table 10:** Perceived basis for discrimination experienced during last labour and delivery

Variable	Frequency (31)	Percentage (%)
Age		
Yes	7	22.6
No	24	77.4
Marital status		
Yes	4	12.9
No	27	87.1
Educational level		
Yes	6	19.4
No	25	80.6
Economic status		
Yes	12	38.7
No	19	61.3
Ethnicity		
Yes	5	16.1
No	26	83.9
HIV/AIDS status		
Yes	2	6.5
No	29	93.5

Table 11 summarises the suggestions by respondents on improvement of maternity services in three areas: ANC services, delivery services and attitude of staff. In the three areas, 20 to 25% of respondents suggested promptness of services, improved care and increased staffing as ways to improve maternity services.

**Table 11:** Suggested improvement to maternity services

Variable	Frequency (384)	Percentage(%)
ANC services		
Be Prompt	25	6.6
Improved care	41	10.7
More staff	31	8.1
No suggestions	287	74.7
Delivery services		
Be Prompt	19	4.9
Improved care	37	9.6
More staff	17	4.4
No suggestions	311	81.0
Attitude of staff		
Be Prompt	9	2.3
Be kind	77	20.1
No suggestions	297	77.5

## Discussion

As demonstrated by this study, obstetric abuse is a reality that occurs against women delivering in identified facilities in Ile-Ife that might hinder achieving the coverage goals for reducing maternal mortality. This study found the prevalence of disrespect and abuse during childbirth in a local government area in Ile-Ife to be 19%, similar to findings in other studies conducted in Tanzania [12] and Kenya [6] reporting a prevalence of 19.5 and 20% respectively. However, a study conducted in Enugu, South Eastern Nigeria [10] reported a prevalence of 98 % suggesting a possible regional or cultural link with respectful maternal care.

This prevalence was despite a high level of awareness by respondents of abuse during childbirth being culturally unacceptable (97.0%), a finding in congruence with Nigeria’s adopted National Standard of Care for Public health facilities, the Charter on Universal Rights of Childbearing Women which does not condone disrespect and abuse in childbearing [2]. An even higher proportion (98.4%) considered respectful and dignifying maternal care a right during childbirth, again, a typical finding in other studies where respectful maternal care is considered a fundamental right of women [13, 14].

Just over 10% of respondents reported knowing someone who had been abused during childbirth which is much lower than reported by midwifery students in Ghana who reported witnessing obstetric abuse at a much higher rate (78.0%) in the maternity rooms [9]. This difference may be due, in part, to the proximity of midwifery students to the childbirth process and low reporting by women who may consider the process as normal or try to move on from an unpleasant experience.

This study found non-dignified care to be the highest form of abuse reported at 12.8% and this was typified, from the opinion of respondents, as being shouted at, use of harsh words or tone or being insulted during the birth process. Present but much less reported was intentional humiliation and threats. Because treatment with dignity determines to a great extent utilization of these facilities, women who have received non-dignified care may prefer to deliver elsewhere. Being discriminated against on account of socio-demographics like economic status, age, educational level, ethnicity, marital and HIV status was also a key finding in this study which is supported by findings in nearby Ibadan, Nigeria that found an association between experience of violence and socioeconomic status [15], however, Innocent et al. in Enugu, Nigeria did not find any significant association between maternal socio-demographic and disrespectful maternal care. Despite being reported as a distasteful practice in facility-based delivery [4, 10], abandonment was also reported by 6% of the respondents, generally as being left unattended or denied necessary care with fewer reports of lack of encouragement during delivery and detainment on account of inability to pay for services. Non-confidential care, which is an unacceptable breach of the code of ethics in healthcare, was found in this study to include being asked private questions publicly, physical examination without a screen and delivery in public view. The report of non-consented care due to inadequate information about care and procedures was low and this may be as a result of increasing awareness among healthcare providers that non-consented care may result in litigation. Physical abuse, however, was least reported by respondents (1.6%) mainly as being pinched, beaten, slapped or sexually abused, however, a single occurrence of sexual abuse is an important issue which needs to be highlighted. These findings are proportionately similar to findings in Kenya [8] and Tanzania [12, 16] though findings from this study seem significantly less than those found in these climes, again likely due to a rising awareness of litigation among care providers.

Respondents in this study suggested kindness from care providers and prompt service as a recipe for improving maternal services, particularly antenatal and delivery services, which are important ways of not stereotyping violence as a part of obstetric care as suggested in other studies [14]. Increased staffing was also suggested, in keeping with identified policy approaches to reducing disrespectful maternal care [2, 4].

Exit interviews were conducted for women who had live born children and were attending postnatal clinics or who brought their children for immunization. Possible limitations of this includes exclusion of the experience of mothers who had stillbirth or and early neonatal death, recall bias and social-desirability bias whereby respondents are likely to give information considered favourable. Another bias which could have occurred is in relation to recruitment (selection) bias as some of the respondents were recruited from postnatal clinics which mothers who may have encountered disrespect and abuse during their deliveries may be unwilling to attend. As women deliver in both formal and informal delivery facilities in Ile-Ife, the demographics of women attending the clinics selected may not completely reflect the demographics of pregnant women in city, and this is a possible limitation to the generalizability of the study. Also, in considering disrespect and abuse as a single item, this conflation may have a possible effect on study validity. A qualitative study could also have been conducted to provide in-depth information regarding the topic beyond the a priori list of categories of disrespect and abuse that were used for the survey. Lastly, discarded responses from the few respondents who were rushing to leave the clinic after receiving services may also pose a limitation.

## Conclusion

This survey revealed varying forms of the occurrence of disrespect and abuse during childbirth in health facilities in this community. It adds to the growing evidence which reveals that poor access to health care services might also be as a result of treatment received by women in these facilities and draws attention to the need for interventions to address the system factors hindering health services utilization.

## Abbreviations

AIDS: Acquired Immune Deficiency Syndrome
ANC: Ante-Natal Care
HIV: Human lmmuno-deficiency Virus
RMC: Respectful Maternal Care
SPSS: Statistical Package for Social Sciences
TRAction: Translating Research into Action
USAID: United States Agency for International Development
WHO: World Health Organization
